# Effect of maternity waiting homes use on maternal and perinatal birth outcomes and its challenges in Amhara region, Northwest Ethiopia

**DOI:** 10.3389/fgwh.2022.978486

**Published:** 2023-01-04

**Authors:** Muluye Molla, Mulugeta Tesfa, Atsede Alle, Firehiwot Molla, Abiot Aschale, Bekalu Endalew, Wodaje Gietaneh

**Affiliations:** ^1^ Health Services Management and Health Economics Unit, Department of Public Health, College of Health Sciences, Debre Markos University, Debre Markos, Ethiopia; ^2^ Epidemiology and Biostatistics Unit, Department of Public Health, College of Health Sciences, Debre Markos University, Debre Markos, Ethiopia; ^3^ Reproductive Health Unit, Department of Public Health, College of Health Sciences, Debre Markos University, Debre Markos, Ethiopia; ^4^ Diseases Prevention and Health Promotion Unit, Department of Public Health, College of Health Sciences, Debre Markos University, Debre Markos, Ethiopia

**Keywords:** effect, maternity waiting homes, maternal outcome, birth outcome, challenge, Amhara region, Ethiopia

## Abstract

**Background:**

Women's death due to complications of pregnancy and childbirth is still high. Maternity waiting homes are one of the strategies to reduce it. However, there is limited evidence on the effect of using maternity waiting homes on birth outcomes, particularly in this study area. Therefore, this study was aimed to estimate the effect of staying in maternity waiting homes use on maternal and perinatal birth outcomes and its challenges in the Amhara region, Northwest Ethiopia 2018.

**Methods:**

Institutional-based comparative cross-sectional study using both quantitative and qualitative approaches was conducted. Data were collected using structured questionnaire interviews, in-depth interview and chart reviews. Propensity score matching analysis was used to estimate the effect of maternity waiting homes use on birth outcomes. Propensity score matching analysis was used to match potential differences in background characteristics that affect pregnancy outcomes between comparison groups. We used thematic analysis for qualitative data.

**Result:**

A total of 548 pregnant mothers (274 stayed in maternity waiting homes 274 did not stay) took part in this study. The proportion of adverse birth outcomes of mothers who stayed in maternity waiting homes were 15(5.5%) which is lower than those who didn't stay 35 (12.8%). After matching with baseline covariates, mean difference of adverse maternal birth outcomes, the difference between didn't use maternity waiting home and used was 10.4%, at (t = 3.78) at 5% level of significance. Similarly, the mean adverse perinatal birth outcomes difference between mothers who didn't use MWHs and used was 11% (t = 4.33).

**Conclusions:**

Maternity waiting home showed a significant positive effect on birth outcomes. Mothers who stayed in the maternity waiting homes had low adverse maternal and perinatal birth outcomes compared to non-users. Accommodations and quality health care services were the challenges mothers faced during their stay in the maternity waiting homes. Therefore, all concerned bodies should give attention accordingly to maternity waiting home services to reduce adverse birth outcomes through the strengthening of the quality of health care provided.

## Introduction

In order to decrease geographic obstacles to get medical care as soon as problems or labor start, the World Health Organization (WHO) has supported the use of Maternity Waiting Homes (MWHs) (1). Maternal waiting home in Ethiopia was initially designed to be utilized by pregnant women at high risk whose home are in isolated, difficult-to-reach rural areas (2).

Currently, regardless of their risk level, maternal waiting rooms are hosting women traveling from rural areas and outside the service delivery region on the last trimester of pregnancy (3). MWHs are therefore a crucial component of a plan to “bridge the geographic gap” in obstetric care between rural areas and those with better access to medical facilities (4).

Worldwide, around 6.3 million live births resulted in death before the age of five (5). The neonatal era saw the deaths of roughly 44 percent (2.8 million) of these youngsters (6). Encouraging expectant mothers to use maternity waiting homes is one strategy to improve the health of newborns (7).

The usage of maternal health care services is poor, and maternal mortality in underdeveloped regions is still 15 times greater than in industrialized regions (8). Every year, pregnancy and childbirth-related problems claim the lives of 20,000 Ethiopian women (9). The intra-partum period surrounding labor and the first postpartum day are when mother mortality peaks (10).

Most of these deaths can be avoided with prompt access to emergency obstetrical care, but the location of the women's residences in relation to the closest medical facility may also have an impact (11). The availability of MWHs lowered the geographic barrier preventing women from accessing skilled care during childbirth (12).

Like other developing nations, Ethiopia launched maternity waiting homes in 1985. There is, however, a dearth of research, especially in our subject area. Additionally, past research did not make an effort to make the group comparable to other factors that influence pregnancy outcomes and instead used straightforward cross-sectional studies. This study evaluated the impact of maternity waiting homes on maternal and perinatal birth outcomes as well as its problems in the Amhara Region, Northwest Ethiopia.

## Methods

### Study design and area

Between September and December 2018, a comparative cross-sectional study situated in an institution and involving 548 rural mothers who gave birth in the East Gojjam Administrative Zone was done. One of the eleven administrative zones in Ethiopia's Amhara National Regional State (ANRS) is East Gojjam Zone.

### Sample size determination and sampling procedures

Utilizing two formulas for population proportions, the sample size was calculated. Using EPI-INFO software version 7.2.4, the total sample size was calculated by taking into account the percentage of stillbirths among mothers who were using the maternity waiting homes setting (P1 = 1.2%), the percentage of stillbirths among non-users of the maternity waiting homes setting (P2 = 10%), the level of significance at the 5% level, and the power at the 80% level (11). Maternity waiting times for MWH users and non-MWH users were 1:1, there was a design effect of 2, and there was a 10% non-response rate. About 548 mothers made up the entire calculated sample size (274 MWH users and 274 non-users). Then, the study participants were selected using a multistage stratified sampling technique. First, among the 20 districts found in East Gojjam zone, seven districts (30% of the study area) were selected by simple random sampling technique namely, Basoliben, Dejen, Hulete Eju Enese, Debre Elias, Enemay and Sinan. Second from each district, two public health centers which have maternity waiting home were selected. Finally, simple random sampling was used to select the study participants. Women who came at the health facility after labor had begun were included, as were mothers who gave birth after staying in maternity waiting homes and mothers who gave birth at the health center directly without using a maternity waiting homes. A total of 18 in-depth interviews with a total of 10 health care providers and 8 mothers were done.

Then, we used a multistage sampling procedure to choose the study participants. First, seven districts—Basoliben, Dejen, Hulete Eju Enese, Debre Elias, Enemay, and Sinan—out of the 20 in the East Gojjam Zone were chosen by simple random sampling to make up 30% of the research area. Second, two public health facilities with maternity waiting homes were chosen, one from each area. Finally, the study subjects were chosen using a simple random sample.

We used the exit interview method to gather information for both mothers who use the maternity waiting home and non-user mothers who give birth in the public health facility.

### Operational definitions

The treatment variable was maternity waiting home use, whereas the outcome variables were maternal and perinatal delivery outcomes (good/poor). Any residence that is close to or is a part of a health facility and is designed for a pregnant woman to stay in before giving birth is known as a maternity waiting home. Any mother death, fistula, uterine rupture, antepartum hemorrhage (APH), postpartum hemorrhage (PPH), and eclampsia are examples of adverse maternal birth outcomes. Any stillbirth, sudden neonatal death, and birth asphyxia are examples of adverse perinatal birth outcomes. Obstacles for mothers: -any social and economic issues mothers' encountered while residing at the maternal waiting home.

### Data collection procedure, data processing and analysis

After reading over a number of pertinent pieces of literature, we created a structured questionnaire. Data were gathered by interviewers using in-depth interview approaches, chart reviews, and administered questions. The data collection includes 14 nurses who served as data collectors and seven nurses who served as supervisors. The data collectors and supervisors received a two-days training on the study's objectives and data gathering methods in order to ensure the accuracy of the data. Investigators oversaw the entire data collection process. To match baseline obstetric and medical characteristics that influence pregnancy outcomes, we used propensity score matching. Finally, using STATA software, we calculated the average treatment effect of treated (ATT) (on average the impact of maternity waiting home stays on maternal and perinatal birth outcomes. A statistically significant influence on the outcomes variables was declared if the t-value was higher than the threshold *p*- value at 0.05. The qualitative results were divided into 6 primary themes and were analyzed thematically.

### Ethical considerations

The ethical review committee of the College of Health Sciences at Debre Markos University granted us ethical approval. Additionally, the East Gojjam Zonal Health Department provided us with letters of support, and each study participant gave their verbal agreement.

## Result

### Socio-demographic and economic characteristics of respondents

We compared the pregnancy outcomes for 274 mothers who delivered at the health center after staying at MWH to pregnancy outcomes for 274 mothers who only arrived at the health center after labor had begun from a total of 548 mothers who participated in this study. All of the participants, 548 (100%) of them were Amhara by ethnicity, had a mean age of 27.5 years (SD + 5.6 years), and 152 (20.7%) of the mothers were Para-I (one) (Table 1).

**Table 1:** Socio-demographic characteristics of the respondents on maternal waiting home utilization, east gojjam, 2017.

Variable (*n* = 548)	Frequency	Percent (%)
Age of the mothers	15–24 years	161	29.4
		25–34	302	55.1
		35–44	82	15.0
		45–49	3	0.5
Marital status	Married	536	97.8
		Single	7	1.3
		Divorced	5	0.9
Religion	Orthodox	529	96.5
		Muslim	19	3.5
Education of mothers	Unable to read and write	500	99.1
		Able to read and write	48	0.9
Mothers' occupation	Farmer	445	81
		Merchant	75	13.7
	Daily laborer	15	2.7	
	Housewife	13	2.4	
Parity of the mothers	Para-I	152	27.7
		Para-II	150	27.4
		Para-III	111	20.3
		Para four and above	135	24.6
Time to start first ANC visits on the current pregnancy	< = 16 weeks	246	44.9
	>16 weeks	302	55.1	

### The proportion of adverse maternal and perinatal birth outcomes among non-MWHs users and MWHs user mothers

In this study, 55(10.04%) mothers experienced unfavorable maternal delivery outcomes in total. Of the negative birth outcomes recorded, mothers who did not use MWH accounted for 35 (63.6%) of the negative maternal birth outcomes. Compared to the poor maternal birth outcomes seen in mothers who used MWH (20), this was worse (7.31%) (See Table 2).

**Table 2:** Adverse maternal and birth outcomes of the respondents on maternal waiting home utilization, east gojjam, 2017.

Adverse outcomes measures	Total number of cases	Proportion for non-MWHs users frequency (%)	Proportion for MWHs users frequency (%)
Eclampsia	11	7 (2.76%)	4 (1.46%)
Obstructed labor	11	8 (2.92%)	3 (1.10%)
PPH*	22	14 (5.11%)	8 (2.92%)
Tear	5	2 (0.73%)	3 (1.10%)
Uterine rupture	2	2 (0.73%)	0
Post-partum sepsis	4	2 (0.73%)	2 (0.73%)
Stillbirth	29	23 (8.39%)	6 (2.20)
Early neonatal death	8	6 (2.20)	2 (0.73%)
Neonatal sepsis	6	3 (1.10)	3 (1.10%)

* PPH, post-partum hemorrhage.

### Impact of utilizing the maternity waiting homes on birth outcomes

First the propensity score was predicted. To predict the propensity score values for the independent variables Chronic Hypertension, Diabetes Mellitus, HIV status, Cardiac Disease, Anemia, Previous C/S, History of APH, History of PPH (Post-partum Hemorrhage, History of PIHT (preeclampsia/Eclampsia). Logit model was employed after the propensity score value predication of the different matching methods were applied (Annex I). Second Balancing test: The next step in assessing the quality of matching is to perform balancing test that checks whether the propensity score adequately balances characteristics between the mothers using the waiting home and mothers who did not use maternal waiting home. Covariates after matching showed that there is no statistically significant difference between covariate means of using and did not use the waiting home (Annex I).

#### Verifying the Common Support Condition

**Figure 1:**
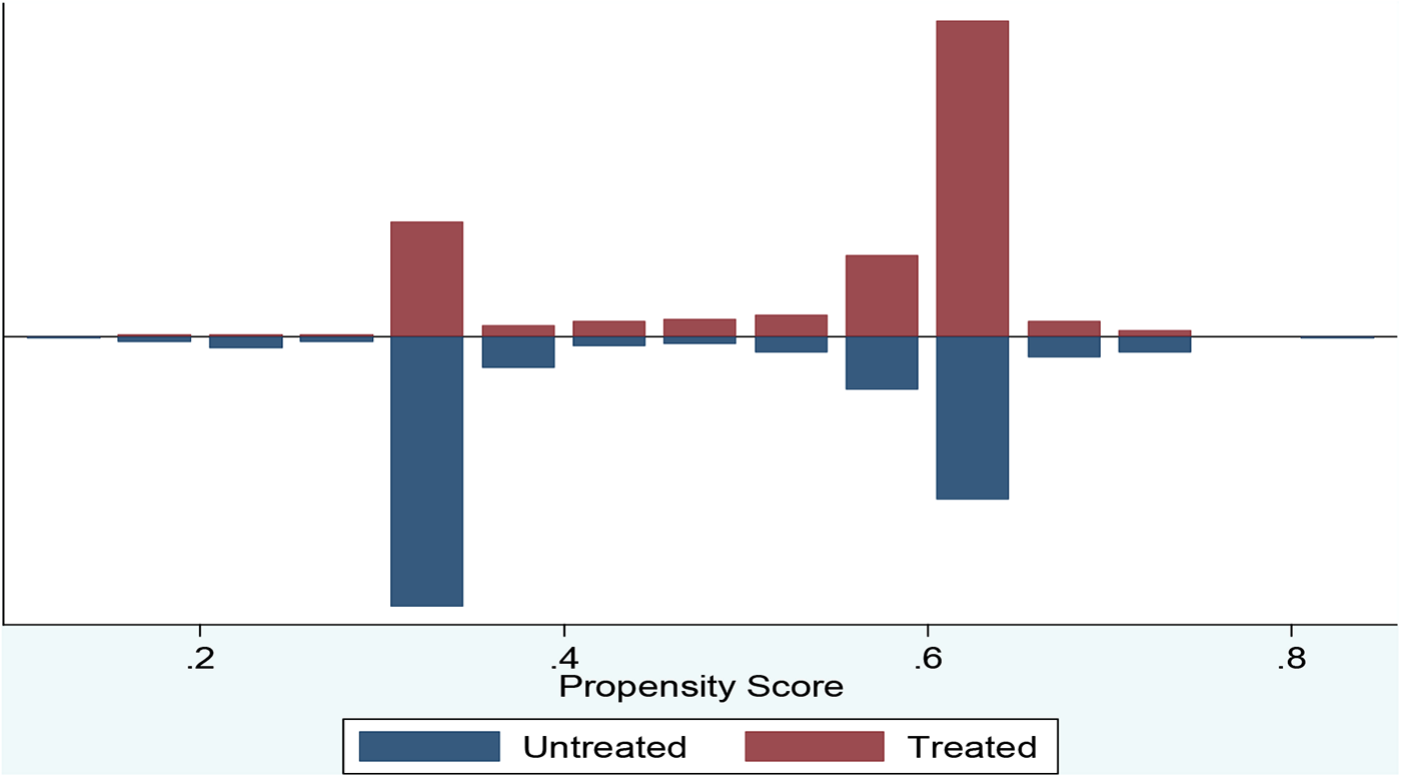
Propensity score matching common support graph on mothers in east gojjam zone, NW Ethiopia, 2017.

In addition to balancing test, another important step in investigating the validity or performance of the propensity score matching estimation is verifying the common support or overlap condition. To demonstrate the common support estimated results and test propensity scores for the two groups of this study, the researcher employed balanced score (PS) graph. The following output shows that the identified region of common support is [.15557549,.74678138] (Figure 1).

After accounting for other variables that influence pregnancy and birth outcomes, mothers who stayed in the waiting homes before giving birth significantly improved the outcomes of birth. Mean good maternal unfavorable birth outcomes differed by 10.4% between users of Waiting Home and non-users (t = 3.78) nearest neighbor matching, at a significance level of 95% (see Table 3).

**Table 3:** Impact of utilizing the maternity waiting homes on birth outcomes among respondents on maternal waiting home utilization, east gojjam, 2017.

Matching method	No of treated	No of controlled	ATT	St. Err	T
Nearest Neighbor	271	252	0.104	0.027	3.78
Radius Matching	275	271	0.096	0.027	3.57
Kernel Matching	275	271	0.099	0.24	4.108

### Impact of maternity waiting home (ATT) on neonatal birth outcome

As shown below (Table 4) mothers` stay in the waiting home before delivery brought the significant positive effect on prenatal birth outcome. After matching other variables that affect prenatal birth outcome, the mothers who stayed in maternity waiting home showed significant positive contribution on good neonatal birth outcome. With mean good neonatal birth outcome difference between waiting home users and not waiting home users mothers in three matching method results that is 11%, 11.1%, 11.2% nearest neighbor matching, radius matching, kernel matching respectively at 5% level of significance.

**Table 4:** The effect of staying in maternity waiting home on prenatal birth out come in east gojjam, NW Ethiopia, 2017.

Matching method	No of treated	No of controlled	ATT	St. Err	T
Nearest Neighbor	271	252	0.110	0.025	4.33
Radius Matching	275	271	0.111	0.025	4.46
Kernel Matching	275	271	0.112	9.25	4.46

## Results of a qualitative study on challenges in maternal waiting home utilization

The qualitative results were divided into 6 primary themes and were analyzed thematically as shown below;

Theme 1: Regarding rooms' space insufficiency and improper preparation:

The in-depth interviewees revealed that the rooms of maternity waiting homes are not properly prepared and insufficient.

“*ክፍሉ አራት አልጋዎች ያሉት ሲሆን ክፍሉ በቂ ስላልሆነ እና ዝቅተኛ በመሆኑ ብዙ ነፍሰ ጡር እናቶች ሲመጡ መጨናነቅ ያጋጥማል። ለሆስፒታል ደንበኞቻችን የተዘጋጁትን ቦታዎች እየተጠቀምን ነው። እናቶች የተለየ መታጠቢያ ቤት ወይም ሻወር መጠቀም አይችሉም። እናቶች የተለየ መታጠቢያ ቤት ወይም ሻወር መጠቀም አይችሉም። እናቶች ለሁሉም ሰው የተዘጋጁ ክፍሎችን ይጋራሉ። እናቶች እዚያ እያሉ ምቾት አይሰማቸውም። በዚህ ምክንያት እናቶች እስኪወለዱ ድረስ እዚህ መቆየት እንደማይፈልጉ አምናለሁ።”* ***(In-depth interviewee 1, A health professional who has been working for four years in institution X)***.

Theme 2: Regarding availability of adequate accommodations in the maternity waiting homes:

*Likewise, in-depth interviewees elaborated that in some institutions, even if the maternity waiting homes' rooms have enough beds and space but* it is not well constructed *and in addition the quality of accommodation services is poor*.

‘‘ ክፍሉ በቂ ቦታ እና አልጋ ቢኖረውም ጥራቱ የተጓደለ እና በደንብ ያልተሰራ ነው። ግድግዳው እና ጣሪያው በቆርቆሮዎች የተገነባ ስለሆነ ክፍሉ ምቹ የሆነ የሙቀት መጠን የለውም፡፡ በሌላ አነጋገር በቀን ውስጥ ይሞቃል፤ በሌሊት ደግሞ በጣም ይቀዘቅዛል. በተጨማሪም የግድግዳው ውስጠኛ ክፍል በ “ቺፑድ” የተሸፈነ በመሆኑ ትኋንና ቁንጫ አልፎ አልፎ ይራባበታል፡፡ ይህ በእናቶች ላይ በሚኖሩበት ጊዜ ተጨማሪ ሥቃይ ያስከትላል፡፡ **(In-depth interviewee 3, a health professional who has worked there for two years in a health institution“Y”)**.

*Theme 3: Regarding access to water* in the maternity waiting homes:

*In some health care facilities, it can be exceedingly difficult to get pregnant women access to water*.

*“በጤና ጣቢያችን ውስጥ ከሚከሰቱት ዋና ዋና ጉዳዮች አንዱ የውሃ እጥረት ነው::ነፍሰ ጡር እናቶች በቂ ውሃ ማግኘት ባለመቻላቸው ንፅህናቸውን ለመጠበቅ አዳጋች ሆኖባቸዋል። ገና የወለዱ እናቶች እንኳን ሻወር አይወስዱም። እናቶች በእነዚህ ክፍሎች ውስጥ መቆየት አይፈልጉም ምክንያቱም ይህ በሚያስከትለው ከፍተኛ ምቾት ማጣት ምክንያት። በእርግጥ ከከተማው “ጄሪካን” በመውሰድ ውሃ ላማቅረብ ይሞከራል, ግን በቂ አይደለም::” **(In-depth interviewee 4, a health professional who has worked three years at institution “Z”).***

Theme 4: Regarding availability of entertainment options in the maternity waiting homes:

As per the in-depth interviewees in most maternity waiting homes there are no entertainment options.

“*ክፍሉ እንደ ቴሌቪዥን ወይም ሌላ የመዝናኛ አማራጮች የሉትም። ለመተኛት, ከጓደኞቻቸው ጋር ለመወያየት ወይም በቡና ሥነ ሥርዓት ላይ ለመገኘት ብቻ ለሚሞክሩ እናቶች, ይህ ችግር ይፈጥራል” **(In-depth interviewee 5, a health expert who has worked for a year in health institution “A”).***

Theme 5: Regarding availability of affordable food in the maternity waiting homes:

According to the in-depth interviewees providing a sufficient and balanced diet for pregnant women who are staying at maternal waiting homes up to delivery and for a few days after delivery is full of difficulties.

“*በተለይ በእርግዝና እና ጡት በማጥባት ጊዜ በቂ እና የተመጣጠነ ምግብ መመገብ ያለውን ጠቀሜታ ለነፍሰ ጡር እናቶች እናስተምራለን። ለምሳ፣ እራት እና ለቁርስ ሰዓት “ሺሮወጥ” በእንጀራ ብቻ እየቀረበላቸው ይመገባሉ።ይህ በቀጥታ የስነ ምግብ ትምህርት ከምንሰጠው ጋር ይቃረናል ምንም እንኳን በቂ በጀት ቢኖርም የህብረተሰቡ የገንዘብ ድጋፍ ቢኖርም የነፍሰ ጡር እናቶችን እና አዲስ የሚወለዱ ህጻናትን ህይወት ማዳን አልቻልንም።* “ *ቁርስ፣ ምሳ እና እራት የሚቀርበው በዚህ ጤና ጣቢያ ነው። ከዚህ ጤና ጣቢያ የሚቀርብልንን እንመገባለን; እኛ መመገብ የምንፈልገውን እንዲቀርብልን ጠይቀን አናውቅም።” **(In-depth interviewee 7, A health care worker who has been employed by institution “B” for one year “; in-depth interviewee 8, A gravida II, para 1 Women in the waiting room of institution “C"”.***

Theme 6: Regarding health professionals’ visit and follow-up in the maternity waiting homes:

*From the moment they enter the waiting area, mothers want regular visits from their health care professionals.* In-depth interviewees' experiences have shown that, due to the lack of medical staff in the health centers, mothers may not attend such institutions frequently unless they have problems. This is taking place since there aren't enough health care personnel and they're operating in separate rooms. Therefore, this can negatively affect pregnant women's perceptions of the maternal waiting homes service. Mothers who do not receive vital sign checks, physical examinations, and other medical attention will not feel psychologically at ease and will believe that it makes no difference if they stay at home until labor begins. Although it is recommended that expectant mothers stay in the maternal waiting home for at least the final two weeks before delivery, the majority of them find it difficult to use the facility due to their primary role in family care/management. They did not go to the MWH as a result, barring significant health issues during her pregnancy. This is one of the main reasons pregnant women don't use maternal waiting homes as much as they should.

“*አልፎ አልፎ እናቶች አንዳንድ ቅሬታ ከሌላቸዉ በስተቀር በሰው ሃይል እጥረት ምክንያት አስፈላጊው የጤና እንክብካቤ በማያቋርጥ መልኩ ክትትል አይደረግም።”*

*“ **A health worker at health center “D”***.

*“በመንገድ መቆራረጥ ምክንያት በቂ የሆነ የትራንስፖርት አገልግሎት የለም። ከእርግዝና ጋር በተገናኘ የጤና ችግር ለሁለት ሳምንታት እዚህ ቆይቻለሁ። በጤና ተቋማቱ ያለው የምግብ አገልግሎት፣የህክምና ስፔሻሊስቶች ክትትል እና ሌሎች ያገኘሁት ሁሉ ጥሩ ነበር ። ይሁን እንጂ ጉዳዩ እኔ ሁልጊዜ ቤቴ የሚኖሩትን የትዳር ጓደኛዬን እና ልጆቼን አስባለሁ, ምክንያቱም ማንም በቤተሰቡ ወይም በመንደሩ ውስጥ ምግብ በማዘጋጀት አይረዳቸውም. በጥሩ ጤንነት ላይ ብሆንም በዚህ ምክንያት ውጥረት ይሰማኛል. ነገር ግን ከዚህ ቀደም ስወልድ ችግር ስለነበረብኝ፣ ባለቤቴ እዚህ እንድቆይ ያበረታታኛል እና ይደግፈኛል።”*

*“**A mother who is para III and gravida IV traveled three hours on foot from her house to institution “E.”***

*Except for mothers who have major health issues and travel from distant locations, expectant women who stay in the waiting house for days desire to go back home if their predicted delivery date is not approaching soon. This is primarily due to their duty to their family. They will not be happy when medical personnel urge mothers to stay until the time of delivery and will go against the recommendation by returning to their house. But there may be other reasons for home delivery besides this*.

*“እናቶች ባሎቻቸውን ወይም ሌሎች ደጋፊ የሆኑ የቤተሰብ አባላትን ወደ አራተኛው የቅድመ ወሊድ ቀጠሮቸው እንዲያመጡ ይመከራሉ ስለዚህም እስከ ወሊድ ድረስ እዚያ በመቆየት ስላለው ጥቅም መወያየት ይችላሉ።” **(a health worker at health center “F,”)***.

*However, they eventually wish to go back to their house when the mother's health improves. There is a chance that they will give birth at home, especially if they go against the advice of medical professionals and return home. This is because they think the doctors won't be delighted to see them again. Therefore, we will inform the health extension workers about mothers who go home again to avoid having a baby at home*.

## Discussion

Reducing maternal and perinatal mortality can be aided by maternity waiting homes (11). According to the results of this study, women who stayed in the MWH before giving birth had a substantial impact on lowering unfavorable maternal and perinatal birth outcomes. When compared to mothers who did not stay in the maternity waiting home, the percentage of negative maternal delivery outcomes was lower among mothers who did not stay.

Mothers of non-MWH users were more likely to experience labor obstruction (2.9% vs. 1 percent). There is no discernible difference between MWH users and non-users in the frequency of uterine rupture, nevertheless. Postpartum hemorrhage (PPH) was more common in mothers who did not stay at the maternity waiting home compared to mothers who did. Early infant death rates were 2.2% (22 per 1000) for mothers who did not stay in MWH against 0.7 percent (7 per 1000) for those who did. Additionally, mothers who did not remain in the MWH had a higher percentage of stillbirths than mothers who did (8.4 percent, 84 per 1,000 vs. 2.1 percent, 21 per 1000).This is consistent with a study conducted in a systemic review in low-income countries and rural Ethiopia (2, 13).

The results of this study demonstrated that staying in maternity waiting homes significantly decreased the likelihood of poor maternal birth outcomes. This is in line with research done in rural Ethiopia (2, 7). This difference in outcomes may be the result of mothers who stayed in the MWH before labor began experiencing shorter delays in the early identification of problems and prompt interventions.

Similar to how the results of this study indicated that using maternity waiting at home before delivery significantly reduced poor perinatal birth outcomes. Compared to mothers who did not stay in the maternity waiting home, mothers who did tended to experience 10% less unfavorable perinatal outcomes. This is consistent with research from a systemic review done in low- and middle-income nations (7). This could be as a result of mothers who did not stay in the maternity waiting homes attempting home birth and visiting medical facilities after complications developed. Overall, maternity waiting homes use leads to better birth outcomes, which is consistent with research done in a nation with limited resources (13). Even though maternity waiting homes have been shown to significantly improve birth outcomes, mothers still faced a number of difficulties while they were there.

The waiting room was insufficient and not prepared to the required standard, according to the qualitative findings. Mothers find it uncomfortable when it gets crowded when they arrive. Mothers were not provided with a separate bathroom or shower. They distributed food from rooms set aside for everyone in the institution. In keeping with a study done in rural Zambia, there was no television in the mothers' waiting area (14). It was extremely difficult for expectant mothers and medical facilities to obtain a balanced diet and enough water. This study agreed with one carried done in Jimma, Southwest Ethiopia (15).

Even in the waiting area, mothers desired frequent visits from their medical professionals. However, the participant's experiences revealed that until mothers complain, it's possible that no one will check on their health. This research is consistent with a study carried out in southern Loa (16).

## Conclusion

Maternity waiting homes had a significant positive contribution to improving maternal and perinatal birth outcomes. Even though maternal waiting homes have significant contributions to the health of mothers and their neonates, mothers faced several challenges during their stay.

## Data Availability

The original contributions presented in the study are included in the article/Supplementary Material, further inquiries can be directed to the corresponding author/s.

## Recommendations

While pregnant women are staying in MWH, special focus should be placed on improving the quality of services provided, and the program needs to be expanded to include eligible mothers. To increase expectant women's use of the MWH, the government shall place appropriate attention, namely on the accessibility of water and suitable human resources in the health institutions. To lessen the difficulties faced by health centers in providing pregnant women with essential consumables while they wait in the waiting room, the government should also alter their financial management practices. The food provided to expectant mothers while they are staying at the maternal waiting homes should be improved to maintain a balanced diet, and the MWH should designate health specialists to provide the expectant mothers with routine checks and care while they are there. It should constantly raise knowledge of the benefits of MWH use in the neighborhood. It would be preferable to conduct extensive research.
